# Renal cell carcinoma presenting with orbital metastasis as the initial symptom: A case report

**DOI:** 10.1097/MD.0000000000048092

**Published:** 2026-03-20

**Authors:** Xuexia Wang, Zhitao Fan, Ranran Liu, Zhenhua Qiao, Wenxin Dong, Sunan Shi, Chaobing Liu

**Affiliations:** a Department of Otorhinolaryngology, Hebei Eye Hospital, Xingtai, Hebei, China.

**Keywords:** differential diagnosis, endoscopic surgery, initial presentation, orbital metastasis, renal cell carcinoma, tumor heterogeneity

## Abstract

**Rationale:**

Orbital metastases are rare, partly because the orbit lacks lymphatic drainage. Metastatic disease initially presenting with isolated ocular symptoms is exceptionally uncommon. We report a case in which ocular manifestations were the first indication of metastatic renal cell carcinoma (RCC).

**Patient concerns:**

An elderly man with no significant prior medical history presented with ocular distension and pain. He reported no systemic symptoms, such as low back pain or hematuria.

**Diagnoses:**

Computed tomography and magnetic resonance imaging revealed a well-circumscribed, round orbital mass with moderate enhancement on contrast-enhanced imaging. Preoperative differential diagnoses included hemangioma and schwannoma. Histopathological examination after surgery confirmed orbital metastasis from RCC. Subsequent abdominal computed tomography identified the primary tumor in the left kidney.

**Interventions:**

Because of the lesion’s proximity to the paranasal sinuses, the mass was completely excised through an endoscopic transnasal approach. The right sinus was entered, the lamina papyracea was exposed, and the tumor was removed completely.

**Outcomes:**

The patient was referred to a comprehensive medical center for further systemic management. At the 1-month follow-up, his ocular symptoms had improved significantly.

**Lessons:**

This case highlights the importance of considering metastatic disease in the differential diagnosis of orbital masses, even in the absence of systemic symptoms. The rarity of orbital metastasis from RCC suggests possible organ-specific metastatic pathways and underscores the need for further investigation into tumor heterogeneity and the molecular mechanisms of site-specific metastasis. Awareness of the diagnostic and management strategies for orbital metastases is clinically important.

## 1. Introduction

Kidney cancer constitutes 5% of all malignant tumors in males, ranking as the sixth most common cancer among men, whereas in females, it constitutes 3% of malignancies and ranks tenth.^[1]^ Renal cell carcinoma (RCC) originates from renal tubular epithelial cells and represents over 90% of renal malignancies.^[2]^ This malignancy demonstrates a distinct predilection for distant metastasis, with preferential dissemination to the lungs, skeletal system, and liver.^[3]^ Although metastatic progression of RCC is thoroughly documented in medical literature, orbital metastases constitute an exceptionally rare clinical entity, accounting for <1% of all orbital tumors. The orbital compartment is considered an unusual site for metastatic dissemination due to its distinctive anatomical characteristics, particularly the absence of conventional lymphatic drainage pathways.

Metastatic orbital tumors often present with nonspecific symptoms such as proptosis, diplopia, or periorbital swelling. However, it is particularly uncommon for isolated orbital symptoms to serve as the sole initial manifestation of an underlying malignancy. In most cases, orbital metastases occur in patients with known advanced cancer. This rare mode of initial presentation, especially when originating from RCC, poses a significant diagnostic challenge for clinicians.

This case report details a unique instance of metastatic RCC in which ocular signs and symptoms were the only initial clinical manifestations, without any accompanying systemic complaints. Through a comprehensive review of the clinical course, imaging findings, and pathological results, this report aims to emphasize the importance of considering metastatic disease in the differential diagnosis of unexplained orbital lesions. Furthermore, we explore the potential implications of this rare metastatic pathway for understanding tumor biology and discuss current management strategies for orbital metastases.

## 2. Case presentation

**Figure 1.**
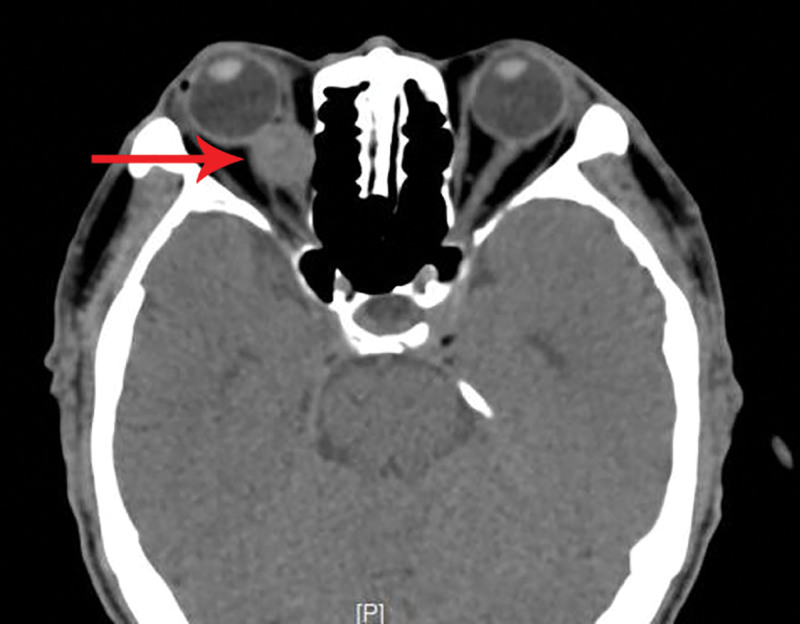
CT reveals a soft tissue mass in the retrobulbar space of the right eye. CT = computed tomography.

**Figure 2.**
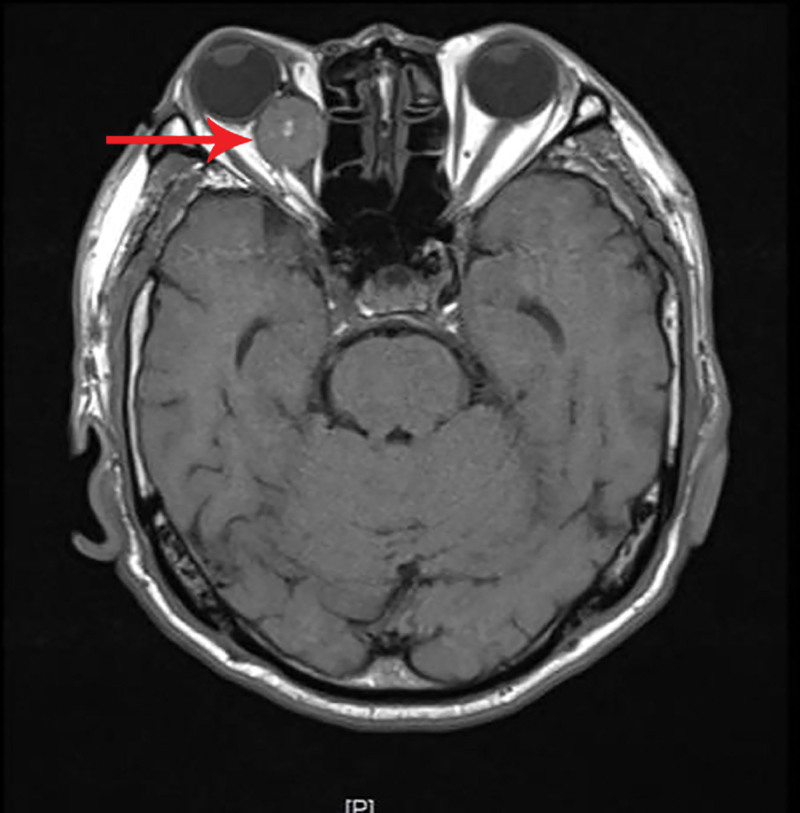
T1-weighted magnetic resonance imaging showing a retrobulbar mass with focal intralesional hyperintensity (arrow).

**Figure 3.**
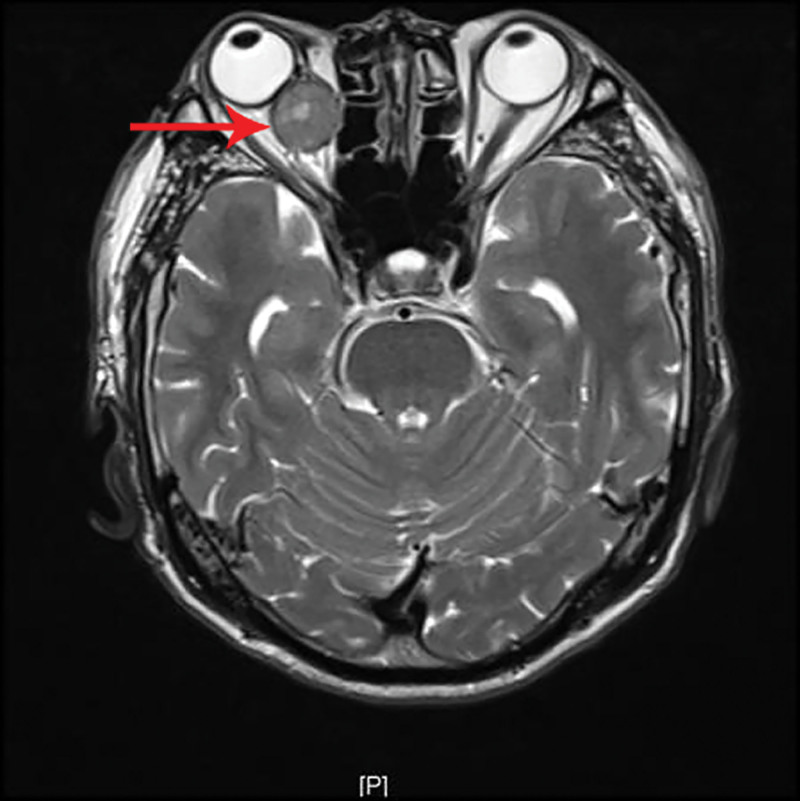
T2-weighted magnetic resonance imaging showing a retrobulbar mass with focal intralesional hyperintensity (arrow).

**Figure 4.**
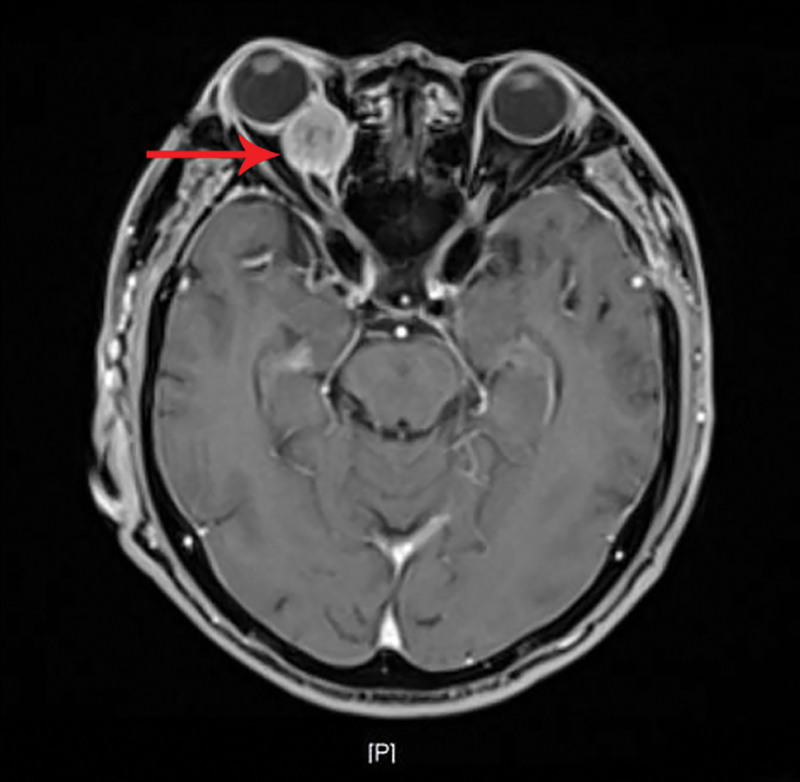
Contrast-enhanced magnetic resonance imaging showing a retrobulbar mass with a rapid wash-in and slow wash-out enhancement pattern (arrow).

**Figure 5.**
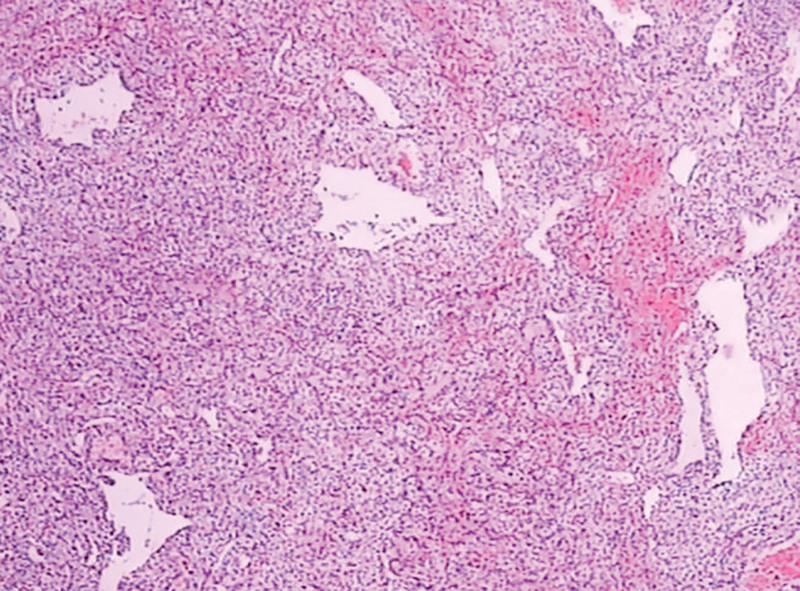
Metastatic carcinoma with solid, glandulopapillary patterns. Tumor cells show abundant, partially clear cytoplasm and atypia in a vascular stroma. (H&E, 200 μm).

**Figure 6.**
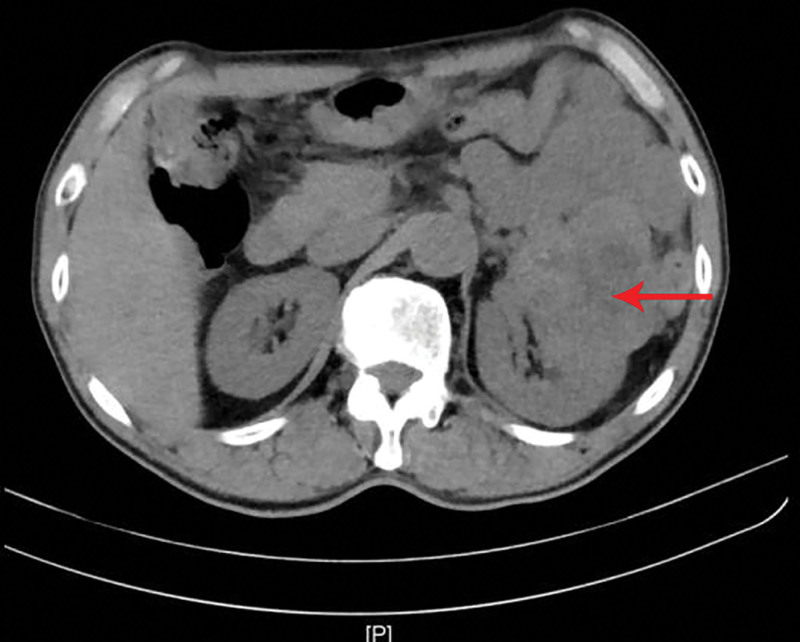
Abdominal CT demonstrates an irregular mass with ill-defined margins and heterogeneous density in the left renal region (as indicated by the arrow). CT = computed tomography.

A 69-year-old male patient was admitted to our department due to right eye distension and pain for 2 months, which had worsened accompanied by proptosis for 1 month. Two months prior to admission, the patient experienced unexplained distension and pain in his right eye. Subsequent orbital computed tomography (CT) revealed a roundish soft-tissue mass lesion in the right retrobulbar fat space. The lesion was poorly demarcated from the medial rectus muscle, measured approximately 14 mm × 16 mm in its largest cross section, and was essentially well-defined. Further investigation was recommended, but the patient declined at that time. One month before admission, the symptoms aggravated, with the emergence of progressive proptosis of the right eye, inferior visual field defect, and diplopia. No headache, dizziness, or other discomforts were reported. Repeat evaluation with orbital magnetic resonance imaging demonstrated a roundish, iso-intense T1 and iso-intense T2 signal mass lesion located postero-nasal to the right globe. A focal area of short T1 and long T2 signal was noted within the lesion. The lesion measured approximately 19 mm × 20 mm × 23 mm and showed moderate enhancement following contrast administration (Figs. 1–4). Hospitalization was advised, and the patient was admitted with a diagnosis of a right orbital mass. On admission, physical examination revealed: best-corrected visual acuity was 0.4 in the right eye and 1.0 in the left eye. Right eye exophthalmos and lateral displacement were observed, with markedly limited adduction. The anterior segment of the right eye was unremarkable. Funduscopy of the right eye revealed retinal folds in the posterior pole. The left eye examination was normal. Visual field testing demonstrated diffuse light sensitivity depression and an inferior visual field defect in the right eye, with an additional localized defect in the superotemporal quadrant. The left eye showed central light sensitivity depression and a localized superotemporal defect. Exophthalmometry measurements were 16 mm for the right eye and 12 mm for the left eye, with an interorbital distance of 103 mm. Pattern visual evoked potentials showed delayed P100 wave latency and slightly reduced amplitude in the right eye, while the left eye results were approximately normal. Chest CT and comprehensive blood laboratory tests showed no significant abnormalities. The patient’s past medical history was unremarkable, with no relevant ocular history or family history of eye diseases. Based on physical examination and ancillary investigations, the right orbital mass demonstrates well-defined borders and a regular morphology, consistent with a benign lesion. A neurogenic or vascular tumor is considered the most likely diagnosis. After completing the relevant examinations, the patient underwent endoscopic transnasal resection of the right orbital mass under general anesthesia. During the surgery, the right sinuses were opened endonasally, the lamina papyracea was fully exposed and removed. The mass was found to be adherent to the medial rectus muscle, encapsulated, and relatively well-defined, and was completely excised. Postoperative pathology confirmed metastatic carcinoma. The tumor tissue exhibited solid nested, adenoid, and papillary structures. The cells had abundant cytoplasm, with some showing clear cytoplasm. The stroma was rich in blood vessels. The cells displayed atypia, and some showed visible nucleoli. Based on the histomorphology and immunohistochemical findings, metastatic RCC was considered. Immunohistochemistry results were: CKpan(+), EMA(+), CAM5.2(+), NSE (partial+), CK18(+), CK20(−), CKLMW(+), CK7(−), CD15(+), CD34 (vascular+), CK5/6(−), Vimentin(+), CD10(+), S-100(−), Syn(−), CgA(−), CD56(−), Ki-67(+) (approximately 30%; Fig. 5). Postoperatively, the patient’s visual acuity gradually improved, with reduction in scotoma. However, exophthalmos and limited adduction persisted in the right eye, while elevation, depression, and abduction were unrestricted. An abdominal CT scan revealed a space-occupying lesion in the left renal region (Fig. 6). The patient was advised to transfer to a general hospital for further diagnosis and treatment; however, due to financial constraints, they declined additional interventions such as surgery, radiotherapy, or chemotherapy. At the 1-month follow-up, there has been significant improvement in right ocular pain and exophthalmos, with limited adduction of the right eye showing slight improvement compared to the immediate postoperative period. No other systemic discomfort is currently reported (Table 1, timeline of key diagnostic milestones).

**Table 1:** Diagnostic and interventional milestones.

Time point	Milestone event	Intervention
Day 0	Reported right ocular distension and pain	–
Day 12	CT scan confirmed a retrobulbar mass as the cause of proptosis and pain	Diagnosed with right orbital mass
Day 60	Retrobulbar mass with classic features: short T1/long T2 signals; rapid wash-in/slow wash-out enhancement	Diagnosed with a benign right orbital mass; hospitalization was advised and arranged
Day 62	Transnasal endoscopic excision of right orbital mass	–
Day 70	Post-op pathology: metastatic renal cell carcinoma. Abdominal CT: occupying lesion, left kidney	Diagnosis: metastatic renal cell carcinoma to the right orbit

CT = computed tomography.

## 3. Discussion

The pathological classification of orbital tumors is relatively complex and can be divided into benign and malignant types. Common benign lesions with relatively high incidence rates include hemangiomas, mucoceles, pleomorphic adenomas, and schwannomas. Malignant tumors are further categorized into primary and secondary orbital tumors. Among primary malignant orbital tumors, adenoid cystic carcinoma and lymphoma have a relatively high incidence.^[4]^ The incidence of secondary malignant orbital tumors is relatively low. Although relevant cases have been reported both domestically and internationally, the reported incidence rates vary slightly.^[5,6]^ According to literature, the most common primary sites for orbital metastatic carcinoma are breast cancer, melanoma, and prostate cancer.^[7-11]^ Other frequently reported cases involve orbital metastases from neuroendocrine tumors.^[12,13]^

RCC is most commonly found in middle-aged and elderly individuals between 50 and 70 years old, with the incidence rate in males being approximately twice that in females. Its incidence is second only to bladder cancer, ranking as the second most common malignant tumor of the urinary system in China. The typical symptoms include hematuria, lumbar pain, and an abdominal mass. The most prevalent pathological subtype is clear cell carcinoma. The most common sites of distant metastasis are the lungs (75%), bones, and liver.^[3]^ However, metastasis of RCC to the orbit is rare, and it is extremely uncommon for orbital metastasis to present as the initial symptom.^[3,6,14-17]^ In a case reported by Cong et al in China, orbital metastasis occurred 1 year after the initial diagnosis of a renal tumor,^[14]^ meaning the ocular symptoms were not the presenting manifestation. There are international reports of RCC where ocular symptoms were the initial presentation.^[15-17]^

In this case, the patient presented with ocular symptoms as the initial manifestation, including eye distension, proptosis, impaired ocular motility, visual acuity, and visual field defects, without typical signs of RCC such as lumbar pain or hematuria. The patient was an elderly male with an orbital tumor that was well-defined and regularly shaped, making it clinically susceptible to misdiagnosis as a primary orbital tumor, particularly a neurogenic or vascular tumor. For elderly patients with unexplained proptosis accompanied by vision loss, it is essential to broaden the scope of differential diagnosis. In clinical practice, careful distinction from other orbital tumors is necessary to avoid misdiagnosis. Given the lesion’s location in the medial orbit adjacent to the paranasal sinuses, the procedure was performed via a transnasal endoscopic approach. The right maxillary sinus, anterior and posterior ethmoid sinuses, and sphenoid sinus were opened to adequately expose the lamina papyracea, which was then removed, allowing complete excision of the mass. This surgical route was chosen due to its advantages: the shortest and most direct access, minimal trauma, lower risk of diplopia and lacrimal duct injury, absence of external skin incision, reduced postoperative pain, and faster recovery. This procedure has benefited from recent advances in endoscopic techniques, minimally invasive philosophy, and oncologic surgery.^[18]^ However, it also has limitations, including the potential for sinus-derived infections and relatively strict requirements regarding tumor location. Postoperatively, the patient’s visual acuity gradually improved, and scotomas reduced. However, proptosis and limited adduction persisted in the right eye, while elevation, depression, and abduction remained unaffected. Postoperative pathological immunohistochemistry confirmed the diagnosis of intraorbital metastatic carcinoma (RCC metastasis). Subsequent abdominal CT imaging revealed a space-occupying lesion in the left renal region. Orbital metastasis from RCC indicates advanced disease with a tendency for systemic dissemination. This necessitates comprehensive systemic evaluation and treatment, along with integrated management to avoid serious complications during therapy.^[19,20]^ Such metastases reflect the highly aggressive biological behavior of RCC, which is likely a major contributor to the poor prognosis. Although malignancies from all primary sites can metastasize to the orbit and lymphatic spread is a common metastatic pathway, the orbit is a relatively uncommon site for distant metastasis due to its lack of a lymphatic system. In most cases, orbital metastases occur in patients with known advanced cancer. It is even rarer for metastatic carcinoma to present with ocular symptoms as the initial manifestation.^[21-23]^ A study has found that high expression of caveolin-1 is positively correlated with the invasiveness and metastatic potential of glioma.^[24]^ The molecular mechanisms underlying distant metastasis in RCC, particularly for rare events such as orbital metastasis, remain incompletely understood. This suggests that tumor cells may favor certain specific pathways to accomplish this process. Beyond the common route of hematogenous spread to intraorbital vasculature, existing research indicates that tumor cells can promote metastasis by remodeling the immune microenvironment of distant organs.^[25]^ Although our findings originate from a specific case, a thorough analysis of each rare case is crucial – viewed from a broader epidemiological perspective – for constructing a comprehensive knowledge framework of such diseases. This approach helps elucidate the underlying mechanisms of tumor heterogeneity and organ-specific metastasis, thereby advancing research into metastatic mechanisms and exploring potential novel therapeutic targets, as supported by macroanalysis of rare tumors.^[26]^

Orbital metastatic tumors often lead to significant functional impairment. The roles of metastatic lesion resection, orbital exenteration, and adjuvant therapy remain controversial.^[27,28]^ Currently, there is a limited number of individual studies on orbital metastatic tumors, and viable treatment options are still debated.^[27,28]^ Treatment strategies depend on the clinical presentation and the pathological nature of the primary tumor; however, a gold standard for treatment has not been established.^[7,29]^ For patients who are not suitable candidates for surgery, orbital radiotherapy may be employed to reduce tumor volume and alleviate symptoms.^[30]^ Surgery can effectively decrease mass effect and improve symptoms but may also lead to serious complications, such as permanent visual deficits.^[31]^ Chemotherapy, hormonal therapy, and/or targeted therapy offer the advantage of simultaneously controlling both primary and metastatic lesions.^[32,33]^ For patients with no history of cancer but clinically suspected orbital or intraocular metastasis, whole-body positron emission tomography/CT scanning is typically recommended. Studies have found that positron emission tomography-CT can detect new metastatic lesions missed by conventional imaging in some patients with metastatic orbital tumors, suggesting its utility in tumor staging and disease burden assessment, as well as in shortening diagnostic time and optimizing systemic treatment plans.^[34-38]^ It is noteworthy that specific primary cancers may exhibit tissue tropism: breast cancer metastases often localize to the orbital fat pad due to the local hormonal microenvironment; melanoma shows a propensity to invade extraocular muscles, manifesting as muscle belly enlargement on magnetic resonance imaging^[39,40]^; while prostate and liver cancers frequently infiltrate orbital bony structures, with osteoblastic/osteolytic reactions clearly observable on CT imaging.^[32,41]^ Survival prognosis depends more on systemic disease control rather than local treatment of the metastatic focus. For patients with systemic malignant tumors, the management of orbital metastases should not necessitate orbital exenteration. Surgical goals should focus on preserving quality of life and avoiding highly debilitating procedures. However, for patients with preexisting severe or complete loss of visual function, orbital exenteration may provide palliative value by alleviating intractable orbital pain.

## 4. Conclusion

Orbital metastatic carcinoma is a rare yet debilitating condition in cancer patients. Histopathological examination is recommended to guide adjuvant treatment strategies. Regardless of the extent of resection, tumor resection appears to be more advantageous than biopsy in alleviating clinical symptoms; however, potential clinical and tumor characteristics should be carefully evaluated on a case-by-case basis when formulating surgical strategies. Orbital exenteration in addition to complete tumor resection appears to offer little additional benefit. Furthermore, orbital radiotherapy is suitable for patients who are not surgical candidates. This article reviews the current status and strategies of systemic and local therapies for orbital metastatic carcinoma through literature analysis, aiming to provide references for clinical practice. However, the conclusions of this study are limited by the short follow-up duration and the lack of detailed systemic treatment data. Prospective studies based on the histopathological characteristics of the primary tumor are warranted to further clarify the role of multimodal systemic treatment strategies in the comprehensive management of orbital metastasis.

## Author contributions

**Data curation:** Zhitao Fan, Ranran Liu, Zhenhua Qiao, Wenxin Dong, Sunan Shi.

**Writing – original draft:** Xuexia Wang.

**Writing – review & editing:** Xuexia Wang, Chaobing Liu.
